# Comparison of Anterior Corneal Curvature Measurements Using a Galilei Dual Scheimpflug Analyzer and Topcon Auto Kerato-Refractometer

**DOI:** 10.1155/2014/140628

**Published:** 2014-05-22

**Authors:** Xiaogang Wang, Jing Dong, Qiang Wu

**Affiliations:** ^1^Department of Ophthalmology, Affiliated Sixth People's Hospital Shanghai Jiao Tong University, No. 600, Yishan Road, Shanghai 200233, China; ^2^Department of Ophthalmology, Shanxi Eye Hospital, Shanxi 030002, China; ^3^Department of Ophthalmology, The First Hospital of Shanxi Medical University, Shanxi 030001, China

## Abstract

*Purpose*. To compare anterior corneal keratometry (K) measurements taken by a dual Scheimpflug analyzer and an auto kerato-refractometer. *Methods*. Sixty-four normal eyes underwent keratometric measurements with both devices. The repeatability of the auto kerato-refractometer measurements was assessed by calculating the coefficient of variation (COV). The interdevice agreement was evaluated using the Bland-Altman analysis, Pearson correlation coefficient, and paired two-tailed *t*-test. *Results*. The COV of the flat *K* and steep *K* measurements taken by the auto kerato-refractometer were 0.21% and 0.29%, respectively. There were no significant differences between the steep *K* and average *K* measurements for the Topcon and Galilei devices (*P* = 0.475, and *P* = 0.137, resp.). The Galilei flat *K* values were lower than those of the Topcon (*P* = 0.002). Both of the instruments showed good agreement for all anterior corneal keratometric values. There was a significant linear correlation between the Galilei and Topcon devices for the flat *K* (*r* = 0.989, *P* < 0.0001), steep *K* (*r* = 0.987, *P* < 0.0001), and average *K* values (*r* = 0.994, *P* < 0.0001). *Conclusions*. The anterior corneal flat keratometric values were not interchangeable between the Galilei and Topcon devices. However, the measurements of the two instruments showed significant linear correlation with each other.

## 1. Introduction

For clinical applications, accurate corneal curvature measurements are important for phakic and aphakic cataract surgery, pre- and postrefractive surgery, and contact lens selection. Therefore, evaluating the instruments that precisely measure anterior corneal curvature is clinically important. Currently, a number of instruments are available for assessing corneal status and measuring corneal curvature, including Scheimpflug topography, optical coherence tomography, optical low-coherence reflectometry, partial coherence interferometry, and slit-scanning topography/pachymetry systems [1–5].

The Galilei Dual-Scheimpflug analyzer (Ziemer Group, Port, Switzerland) is a high-precision optical system for evaluating corneal topography; it is different from the Pentacam (Oculus), a Scheimpflug-based system that derives its surface keratometry readings from Scheimpflug images. The Galilei has the advantages of a revolving dual Scheimpflug camera and a Placido disk. The corneal curvature measurements generated by the Galilei have been shown to have excellent repeatability [6].

The KR-8800 auto kerato-refractometer (Topcon, Tokyo, Japan) uses rotary prism technology to assess corneal refractive status; it measures spherical refractive power, cylindrical refractive power, the direction of the astigmatic axis, corneal curvature, the direction of the principal meridian, and the corneal refractory power. There are few studies about the repeatability of this instrument for keratometry measurement.

The purpose of this study was to compare the anterior corneal curvature measurements of the Galilei and Topcon instruments in normal subjects. We also investigated the repeatability of the corneal curvature measurements obtained using the Topcon instrument.

## 2. Materials and Methods

This study was performed at the Affiliated Sixth People's Hospital* Shanghai Jiao Tong University* (Shanghai, China). The research protocols were approved by the institutional review boards and carried out in accordance with the tenets of the Declaration of Helsinki. Written informed consent was obtained from each subject after they were given an explanation of the nature of the study.

This study included a total of 64 eyes without ocular abnormalities other than cataracts from 64 normal subjects (28 males and 36 females). One eye from each subject was randomly selected. The intravisit repeatability of the Topcon instrument measurements was calculated based on data from 4 sets of consecutive measurements within a single visit for 10 subjects. All 64 eyes were included in the comparison of the corneal curvature measurements performed by the Galilei and Topcon instruments. The data capture procedure for both devices was as follows: the subject's chin was placed on the chin rest, the subject's forehead was pressed against the forehead strap, and the subject's eye was aligned to the visual axis by a central fixation light or target. A single trained operator performed all of the examinations using both instruments following the procedural guidelines for the Topcon and Galilei instruments.

Similar to traditional keratometers, the Galilei (Software Version 5.2.1) uses a hypothetical keratometric index of *n* = 1.3375, which is based on a model of the cornea as a single refracting surface, for the anterior corneal curvature calculation within the region of interest, which is approximately 1–4 mm in diameter. The Topcon KR-8800 enables refraction measurements with a minimum pupil size of 2 mm and probably generates more reliable objective refraction data with its rotary prism technology.

The statistical analyses were performed with commercial software (SPSS ver. 13.0; SPSS Inc.). The repeatability of the auto kerato-refractometer was assessed by calculating the coefficient of variation (COV). The statistical significance of the interdevice differences in corneal curvature parameters was evaluated with the paired two-tailed *t*-test. The correlation coefficient was also calculated, and a scatter plot was created to evaluate the relationship of the corneal curvature measurements between the two instruments. Interdevice agreement was evaluated using Bland-Altman analysis [7]. The interdevice differences were plotted against their means, and the 95% limits of agreement (LoA) were determined using this method. The significance level for all of the tests was set at 5%.

## 3. Results

The mean age of all of the enrolled subjects was 26.3 ± 14.5 years (range, 12–63).  Table 1 shows the mean flat *K*, steep *K*, and average *K* values for each instrument.

There were no significant differences between the Topcon and Galiei instruments for the steep *K* and average *K* measurements (*P* = 0.475, *P* = 0.137). The flat *K* values measured by the Galilei were significantly lower than those of the Topcon (*P* = 0.002).

**Figure 1:**
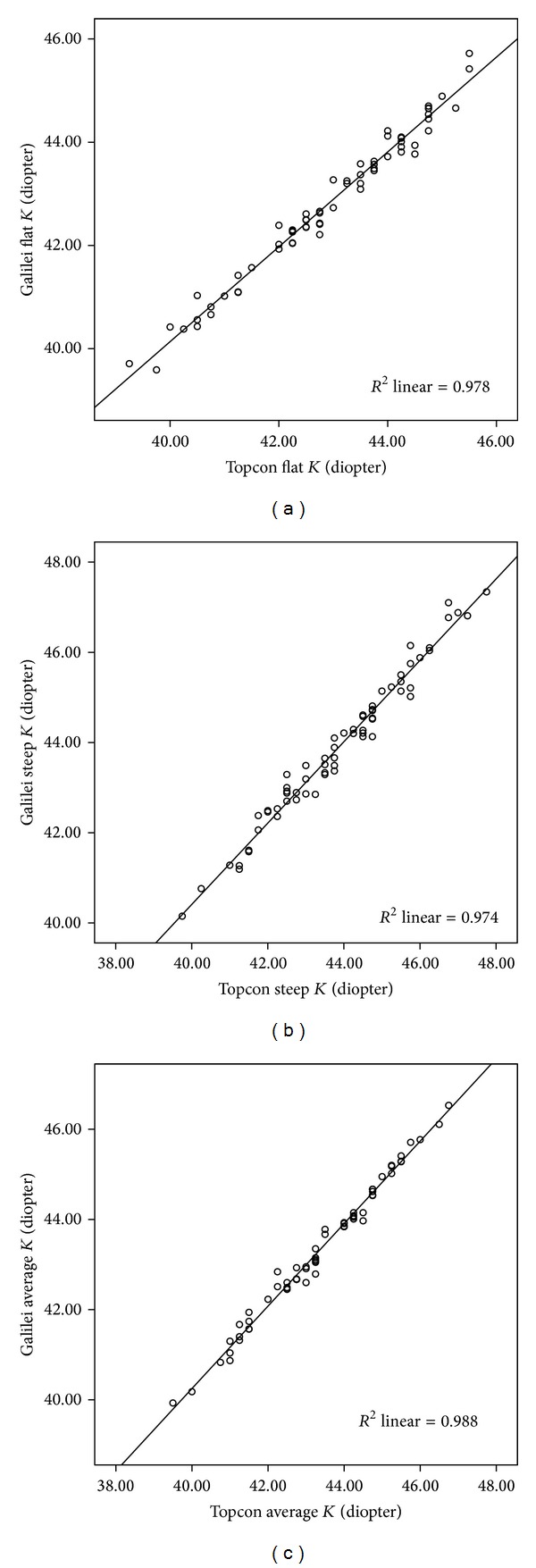
There was a significant linear correlation between flat *K* measurements by Galilei and Topcon ((a) *r* = 0.989, *P* < 0.0001). The best-fit line (*y* = 3.39 + 0.92*x*) is designated by the solid line; there was a significant linear correlation between steep *K* measurements by Galilei and Topcon ((b) *r* = 0.987, *P* < 0.0001). The best-fit line (*y* = 4.33 + 0.90*x*) is designated by the solid line; there was a significant linear correlation between average *K* measurements by Galilei and Topcon ((c) *r* = 0.994, *P* < 0.0001). The best-fit line (*y* = 3.66 + 0.92*x*) is designated by the solid line.

There was a significant linear correlation between the Galilei and Topcon instruments for the flat *K* (*r* = 0.989, *P* < 0.0001) (Figure 1(a)), steep *K* (*r* = 0.987, *P* < 0.0001) (Figure 1(b)) and average *K* values (*r* = 0.994, *P* < 0.0001) (Figure 1(c)).

**Figure 2:**
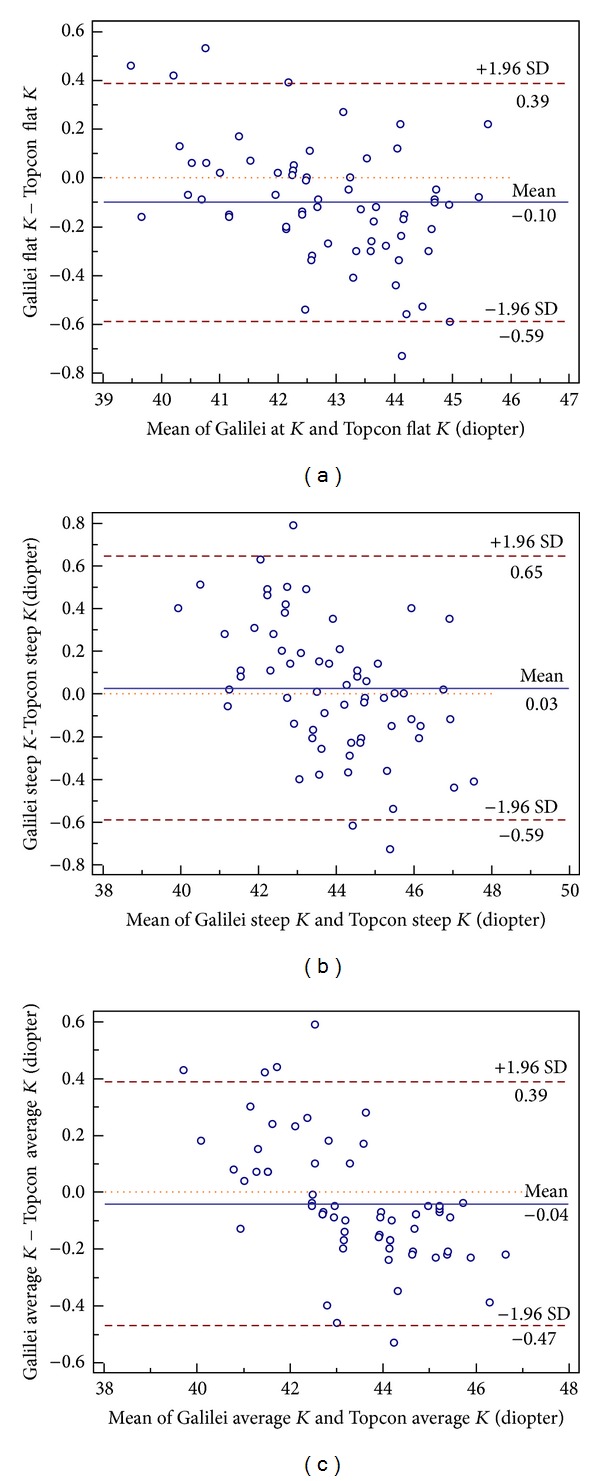
Differences in mean anterior corneal keratometric values between Galilei and Topcon. The means ± standard deviation (SD) are indicated.

Bland-Altman plots were created to evaluate the differences in the individual measurements as a function of the mean of the two instruments for each subject. Both methods showed good agreement for the three anterior corneal curvature parameters that were measured with mean of differences centering around zero (Figure 2). The interdevice 95% LoA ranges for the flat *K*, steep *K*, and average *K* values were 0.98 D, 1.24 D, and 0.86 D, respectively. The difference in the average *K* values between the two instruments showed the smallest range of variation (Figure 2(c)).

Ten normal eyes were scanned to assess the intravisit repeatability of repeated measurements by pooled COV. The repeatability of the Topcon instrument was 0.21% for flat *K* and 0.29% for steep *K*.

## 4. Discussion

The accurate determination of corneal curvature not only is an important factor in the diagnosis and follow-up of keratoconus but also is important in the determination of the eligibility of patients for refractory surgery and the level of correction that can safely be performed during refractive surgery. As a routine examination device for corneal refractive status measurement, the auto kerato-refractometer has been widely used clinically. As a new technology, the Galilei uses a rotating Scheimpflug system in addition to the Placido disk, allowing the corneal posterior surface as well as the anterior surface to be analyzed. Using the Pentacam, which uses a rotating Scheimpflug camera to image and analyze the anterior segment of the eye and a Placido-scanning-slit system (Orbscan II), several previous studies reported similar values for anterior corneal curvature measurements to those we obtained in our normal subjects [3, 8, 9]. However, the corneal curvature values in our study were lower than those published by Liu et al. in a study using Orbscan [4]. Our study showed that anterior flat keratometry measurements are not interchangeable between the Galilei dual Scheimpflug analyzer and Topcon auto kerato-refractometer. Although the reason for this discrepancy is unclear, the distinct methodologies used for each device may explain the difference.

Unlike ultrasound pachymetry, the gold standard for corneal thickness measurement, there is currently no gold standard to test the accuracy and precision of corneal curvature measurements. However, our study measured the intravisit repeatability of the Topcon by taking four consecutive measurements for each patient within one visit and showed excellent repeatability for the Topcon instrument that was as high as that previously reported for the Galilei instrument [6].

This is the first study to compare the corneal curvature measurements between the Galilei and Topcon instruments. Unlike the results of Guilbert et al., which showed that the corneal curvature measurements were interchangeable between the combined Placido-Scheimpflug system and the combined Placido-scanning-slit system, our results showed a significant difference between the Galilei and Topcon instruments in the flat *K* measurement [9].

The Bland-Altman plots show that the 95% LoA for the difference in the flat *K* measurements between the Galilei and Topcon ranged from −0.59 D to 0.39 D, meaning that the Galilei values could be as much as 0.59 D lower than the Topcon values. These discrepancies are likely to be clinically significant. However, the correlation between the two devices was excellent. The Bland-Altman plots for the comparison of the two instruments showed that the differences in the corneal curvature measurements varied with the actual curvature measurement. Therefore, it may be possible to generate an appropriate conversion formula that will allow readings from the two devices to be interchangeable.

One limitation of this study was that we only compared the measurements of these instruments in normal eyes. However, the importance of validating the interchangeability of these values between these two instruments in healthy eyes before investigating their use in corneal disease diagnosis is well known. Therefore, although our present study focused on normal subjects, as no previously published study compared the Galilei system to the Topcon system for corneal curvature measurement, our study has provided important information for clinical applications. Moreover, the drift of measurements between the instruments, which may be caused by the vibration during operation and the signal instability, should be considered. Therefore, the recalibration for each instrument in a routine time is needed in the clinical practice.

In conclusion, this comparative study showed an excellent correlation and good agreement between the Galilei and Topcon instruments for evaluating the anterior corneal curvature in normal eyes. However, the flat keratometry readings measured by the two devices were not interchangeable.

## Figures and Tables

**Table 1:** Summary of anterior corneal curvature measurement and mean interdevice difference between Galilei and Topcon.

	Flat *K* (D)	Steep *K* (D)	Average *K* (D)
Galilei	42.80 ± 1.44	43.92 ± 1.63	43.36 ± 1.51
Topcon	42.90 ± 1.55	43.89 ± 1.78	43.40 ± 1.64
Galilei-Topcon	−0.10 ± 0.25	0.03 ± 0.31	−0.04 ± 0.22
P*	0.002	0.475	0.137

*Paired sample *t*-test.

D: diopter.
